# Chaperoned amyloid proteins for immune manipulation: α-Synuclein/Hsp70 shifts immunity toward a modulatory phenotype

**DOI:** 10.1002/iid3.39

**Published:** 2014-12-05

**Authors:** Adahir Labrador-Garrido, Marta Cejudo-Guillén, Rebecca Klippstein, Erwin J De Genst, Laura Tomas-Gallardo, María M Leal, Javier Villadiego, Juan J Toledo-Aral, Christopher M Dobson, David Pozo, Cintia Roodveldt

**Affiliations:** 1 CABIMER, Andalusian Center for Molecular Biology and Regenerative MedicineSeville, Spain; 2 Department of Medical Biochemistry Molecular Biology and Immunology School of Medicine, University of SevilleSpain; 3 Department of Chemistry, University of CambridgeUK; 4 CABD, Andalusian Center for Developmental BiologySeville, Spain; 5 IBiS Institute of Biomedicine of Seville, University Hospital Virgen del Rocío-CSIC-University of SevilleSpain; 6 Department of Medical Physiology and Biophysics School of Medicine, University of SevilleSpain; 7 CIBERNED, Centers for Networked Biomedical Research in Neurodegenerative DiseasesSpain

**Keywords:** amyloid disease, heat-shock proteins (HSPs), immunization, immunomodulation, α-synuclein

## Abstract

α-Synuclein (αSyn) is a 140-residue amyloid-forming protein whose aggregation is linked to Parkinson's disease (PD). It has also been found to play a critical role in the immune imbalance that accompanies disease progression, a characteristic that has prompted the search for an effective αSyn-based immunotherapy. In this study, we have simultaneously exploited two important features of certain heat-shock proteins (HSPs): their classical “chaperone” activities and their recently discovered and diverse “immunoactive” properties. In particular, we have explored the immune response elicited by immunization of C57BL/6 mice with an αSyn/Hsp70 protein combination in the absence of added adjuvant. Our results show differential effects for mice immunized with the αSyn/Hsp70 complex, including a restrained αSyn-specific (IgM and IgG) humoral response as well as minimized alterations in the Treg (CD4^+^CD25^+^Foxp3^+^) and Teff (CD4^+^Foxp3^−^) cell populations, as opposed to significant changes in mice immunized with αSyn and Hsp70 alone. Furthermore, in vitro-stimulated splenocytes from immunized mice showed the lowest relative response against αSyn challenge for the “αSyn/Hsp70” experimental group as measured by IFN-γ and IL-17 secretion, and higher IL-10 levels when stimulated with LPS. Finally, serum levels of Th1-cytokine IFN-γ and immunomodulatory IL-10 indicated a unique shift toward an immunomodulatory/immunoprotective phenotype in mice immunized with the αSyn/Hsp70 complex. Overall, we propose the use of functional “HSP-chaperoned amyloid/aggregating proteins” generated with appropriate HSP-substrate protein combinations, such as the αSyn/Hsp70 complex, as a novel strategy for immune-based intervention against synucleinopathies and other amyloid or “misfolding” neurodegenerative disorders.

## Introduction

α-Synuclein (αSyn) is a highly conserved, soluble protein which is abundant in various regions of the brain, and which is currently believed to play a role in modulating synaptic plasticity, neurotransmitter release, and presynaptic vesicle pool size 1–3. However, the aberrant misfolding and aggregation of this protein and, ultimately, its conversion into insoluble amyloid-like fibrils, are linked to Parkinson's disease and other synucleinopathies 4. These and other “misfolding/conformational” disorders are characterized by conversion of an initially soluble and functional polypeptide into aggregation intermediates to ultimately produce insoluble aggregates that can be structurally amorphous or fibrillar 5. In each particular disorder, unfolding and/or misfolding of specific proteins (for simplicity, herein referred to as “aggregating proteins”) are key initial steps in the aberrant aggregation and amyloid formation process 6.

Like many of the peptides and proteins that are involved in the most common misfolding and amyloid diseases – including Alzheimer's, Huntington's, type II diabetes, and spongiform encephalopathies – αSyn is an intrinsically disordered protein (IDP) in its free soluble form 6. IDPs are a group of polypeptides that lack significant secondary and tertiary structure, as well as many specific intra-chain interactions 7. The high degree of conformational flexibility in IDPs has been suggested to underlie the observations that these proteins upon immunization tend to produce weak immune responses 8 and that they help certain pathogens escape immune detection by the host 9.

In the last decade, a link has been established between αSyn and the abnormal immunological process that accompanies the onset and progression of synucleinopathies 10,11. In addition to a robust microglia activation and sustained neuroinflammation in the brain, changes in the T cell-mediated immunity in the brain and the periphery – including an increased presence of T effector (Teff) memory cells and dysfunctional regulatory T (Treg) cells – are seen during PD progression 12. In the case of the synucleinopathies, several studies performed in animal models have found that CD4^+^ T cells are critically involved in the neurodegenerative and neuroprotective antagonistic processes associated to disease 13–17. Based on this evidence, it has been proposed that identifying modulators that are able to restore the imbalance in the Treg-/Teff-mediated immunity and to induce a regulated, “neuroprotective” immunological environment, might be the key for developing effective immunotherapeutic strategies against neurodegenerative disorders 16,18,19.

Several studies carried out following the pioneering work by Masliah et al. 20 have explored different types of approaches to immunization with α-synuclein in animal models, which have produced promising albeit mixed results, or with as yet uncharacterized therapeutic efficacy in human subjects 13,14,20–24. Therefore, the development of novel αSyn-based vaccination strategies for synucleinopathies and related disorders stands as a highly attractive but challenging avenue for research and development.

In addition to the long-established chaperoning functions of heat-shock proteins (HSPs) including binding to, remodeling, and conformational stabilization of, unfolded/misfolded client polypeptides 25, certain HSPs have been increasingly reported to play diverse roles as modulators of the innate and adaptive immunity 26–29. Such functions range from promoting antigen cross-presentation and the maturation of dendritic cells, to exerting immunosuppressive signals, or facilitating the activation of lymphocytes and macrophages 30–33.

Therefore, HSPs possess two highly valuable features that could be simultanously exploited for aggregating protein-based manipulation of the immune response against misfolding or amyloid disorders, namely, their classical “chaperone” functions combined with their recently discovered “immunoactive” properties. Indeed, this second property has been used to boost the antibody production by immunizing mice with DnaK (bacterial) HSP chemically cross-linked with Aβ peptide 34 and PrP protein 35, and with a 17-aminoacid sequence from Hsp60 conjugated to an Aβ epitope 36, that is by utilizing either inactivated HSP proteins or HSP short fragments, as carriers. Still, to the best of our knowledge, no comprehensive characterization of the immune response elicited by an “HSP-chaperoned aggregating protein” complex – that is a complex generated with a “functional chaperone” able to interact productively with the aggregating protein substrate and display its full biological activities – has thus far been reported. In this study we decided to investigate, as a proof-of-concept, the immune response elicited by immunization with a complex-forming HSP-αSyn combination. We took advantage of the acquired knowledge on Hsp70, a highly conserved HSP 37, which has been shown to bind to αSyn aggregation intermediates 38–41 and to interact with αSyn monomers 40. We report a differential immunological profile as a result of immunizing naive mice with a combination of highly purified human Hsp70 and α-synuclein proteins in the absence of added adjuvant.

## Materials and Methods

### α-Synuclein and Hsp70 protein overexpression, purification, and characterization

Human Wt αSyn was over-expressed in *Escherichia coli* BL21(DE3) cells using pT7-7 plasmid and purified as described previously 42. The purity and monomeric state of the αSyn protein preparation (>95%) were assessed by 15% SDS-PAGE, 4–12% native PAGE (Lonza, Basel, Switzerland), and mass spectrometry (not shown), as previously described 42. Recombinant N-hexa-His-tagged human Hsp70 (HSPA1A), which was previously cloned into the pET28b vector (Novagen, Merk Millipore, Darmstadt, Germany) was overexpressed in *E. coli* BL21(DE3) (Lucigen, Middleton, WI, USA) and then purified and treated as described previously 40. The purity of the Hsp70 preparation (>95%) was assessed by 12% SDS-PAGE. After passing the protein solution through a Amicon Ultra-100 kDa (Merck Millipore Ltd., Carrigtwohill, IRL), the protein was assayed for its endotoxin content by the ToxiSensor Chromogenic LAL Assay Kit (GenScript, Piscataway, USA). The endotoxin levels of the protein preparations were <1 EU/mg protein in all cases. Protein concentrations were determined by means of Micro BCA Reagent Kit (Pierce, Rockford, IL, USA).

### Preparation of the αSyn/Hsp70 complex

In order to favor the formation of the αSyn/Hsp70 complex, the purified αSyn and Hsp70 proteins were pre-incubated at a 1:1 molar ratio in “Hsp70 buffer” (50 mM Tris/HCl pH 7.4; 150 mM KCl, 2 mM MgCl_2_) in the presence of 4 mM adenosine 5′-triphosphate magnesium salt (ATP) (Sigma–Aldrich, St. Louis, USA) for two hours at room temperature (RT), after which time adenosine 5′-diphosphate monopotassium salt dehydrate (ADP) (Sigma–Aldrich St. Louis, USA) was added to a 2.5 mM final concentration and incubated for a further two hours at RT. Sample preparations consisted of αSyn alone, Hsp70 alone, a mixture of both, or Hsp70 buffer, and they all contained the same buffer and received the same incubation treatment. For immunization purposes, samples were diluted accordingly in PBS after incubation.

### Western blot assay for αSyn/Hsp70 complex characterization

In order to assay the formation of the αSyn/Hsp70 complex, protein preparations were loaded onto a 4–12% native PAGE (Lonza, Basel, Switzerland) and subjected to electrophoresis at 120 V, and transferred for 45 min onto 0.2 µm nitrocellulose membrane (GE Healthcare, Buckinghamshire, UK). After blocking overnight with 5% skimmed milk in PBST (0.05% Tween 20 in PBS), the membranes were probed with the mouse anti-α/β-synuclein (N19) polyclonal antibody (Santa Cruz Biotechnology Inc. Heidelberg, Germany) or the anti-Hsp70 monoclonal antibody (C96F3-3) (Enzo Life Sciences inc. Farmingdale, NY, USA). HRP-conjugated anti-goat (Santa Cruz Biotechnology inc. Heidelberg, Germany) and anti-mouse (Promega, Madison, WI, USA), secondary antibodies were used to visualize blots by using Immobilion™ Western Chemiluminiscent HRP Substrate (Millipore, Billerica, MA, USA) and AmershamHyperfilm™ ECL (GE Healthcare, Buckinghamshire, UK).

### Surface plasmon resonance detection of α-synuclein-Hsp70 interaction

Surface plasmon resonance experiments were performed in a Biacore X100 instrument with a CM5 sensor chip (GE Healthcare). 50 nM Hsp70 (ligand) was immobilized through the amine coupling chemistry, as follows. Both flow cells were activated for 7 min with a 1:1 mixture of 0.1 M *N*-hydroxysuccinimide (NHS) and 0.4 M 3-*N*,*N*-dimethylamino (EDC) at a flow rate of 5 µL/min. Immobilization was performed in sodium acetate buffer (pH 5.0). The ligand (originally in a solution in “Hsp70 buffer”) was injected at 5 µL/min in 10 mM sodium acetate buffer (pH 5.0) at a concentration of 480 nM on the activated sensor surface 2 (Fc2) and then 1 M ethanolamine-HCl (pH 8.5) was added to block the unreacted *N*-hydroxysuccinimide groups. The level of immobilized ligand was 3173 response units (RU). The sensor surface 1 (Fc1) was activated/deactivated without ligand for its use as reference surface for subtraction of non-specific signal effects. The running buffer for all the experiments was 1× PBS supplemented with 0.05% Surfactant P20, 2 mM MgCl_2_, and 2.5 mM adenosine 5′-triphosphate disodium salt (ATP). All samples were diluted in running buffer at 0, 20, 40, 80, 160, and 320 µM, in increasing concentrations order with at least one duplicate of lower concentration after the highest analyte concentration. Contact time for binding and dissociation time were 300 sec. All the binding cycles were performed at 15°C. No regeneration was applied to the sensor surfaces as sensorgrams readily returned to the baseline in the dissociation phase. An extra wash with running buffer was applied at the end of each cycle.

Interaction data were analyzed using the Biacore X100 Evaluation Software (GE Healthcare). The equilibrium dissociation constant (*K*_D_) was calculated from steady-state sections of the curves 5 sec before analyte injection stop, by using the Affinity Wizard tool.

### Animals

Six to seven week old, C57BL/6 male mice were purchased from the University of Seville Center for Animal Production and Experimentation (Espartinas, Spain). Animals were kept for one week in the local animal house before the start of the immunization protocol, to allow the mice to acclimatize to their new environment. At all stages of the study, animals from each experimental group were allocated into different cages such that each cage contained up to five mice, in every case corresponding to a mix from different experimental groups. All animal procedures were in accordance with good animal practice as defined by the relevant national/EU and ARRIVE guidelines and the CEEA-CABIMER Experimental Animal Committee, and all animal procedures were approved by the corresponding committee (CEEA-2010-14).

### Immunization protocol

Mice were immunized on day 0 with 5 or 50 µg of Hsp70 (“low” or “high” dose, respectively) and/or 1.06 or 10.6 µg of αSyn (“low” or “high” dose, respectively). All preparations were diluted in PBS (ca. 50-fold dilution of the pre-incubation mixtures) in the absence of added adjuvants. Mice were injected with a single 100 µL s.c. shot in the lumbar region. The same procedure was repeated on day 7. On day 14, mice were sacrificed, and the spleen and 500–700 µL of blood, were extracted for analyses.

### Determination of CD4+, Treg and Teff cell populations

Splenocytes were isolated from the spleen of immunized mice by perfusing with 10 mL with PBS after which erythrocytes were lysed by osmotic shock. The number of cells was determined by counting them in a hemocytometer and 10^6^ cells were labeled with anti-CD4-FITC, anti-CD25-APC, and anti-Foxp3-PE, antibodies (BD Biosciences, San Diego, CA, USA), by following the manufacturer's instructions. Flow cytometry analysis was performed with FACS Calibur cytometer and CellQuest Pro (BD Biosciences, San Diego, CA, USA) software.

The Treg cell population was calculated as the percentage of cells positive for CD4, CD25, and Foxp3 staining among the CD4^+^ lymphocyte population. The Teff (non-regulatory) cell population was calculated as the percentage of cells that stained positively for CD4 and negatively for Foxp3 from the CD4^+^ lymphocyte population (Supporting Information Fig. S1).

### In vitro stimulation of splenocytes and determination of secreted cytokines

Splenocytes were isolated from the spleen of immunized mice after sacrifice, as previously described. 3 × 10^6^ cells from each mouse were divided and cultured in three wells (of a 12-well plate) in RPMI medium (BioWhittaker, Verviers, Belgium) with 10% inactivated fetal bovine serum (FBS, BioWhittaker, Verviers, Belgium). Each well was treated as follows: well 1, medium alone (control); well 2, αSyn (20 µg/ml); well 3, lipopolysaccharide from *Escherichia coli* serotype 0127:B8 (LPS) (Sigma–Aldrich, St. Louis, USA) (0.5 µg/mL). After incubation for 24 h, supernatants were collected and centrifuged at 500 *g* for 5 min to eliminate any remaining cells and debris, and stored at −80°C for subsequent cytokine assaying.

For quantifying IFN-γ, IL-10, and IL-17 levels from culture supernatants, specific ELISA kits, namely Mouse IFN-gamma and Mouse IL-10 BD OptEIATM kits (BD Biosciences, San Diego, CA, USA), and ELISA Development Kit Murine IL17 (PreproTech, London, UK), were used according to the manufacturer's instructions.

### Antibody content and cytokine measurement in mouse sera

Blood samples extracted after sacrifice were left for 1 h at 4°C and 1h at RT to let them clot. After clot formation samples were centrifuged at 21,000 *g* for 15 min to obtain cell-free serum, and stored at −80°C for further analyses.

To assay the content of total IgM and IgG antibodies, samples were diluted 1:240,000 in PBS and 100 µL aliquots were transferred to a 96-well plate well (MaxiSorp plate, NUNC, Roskilde, Denmark) and incubated for 1 h at 37°C. Next, wells were washed three times with 350 µL of PBST (PBS, 0.05% Tween20) and blocked for 1 h with Assay Diluent (BD Biosciences, San Diego, CA, USA). After washing as in the previous step, wells were incubated for 1 h at 37°C with anti-IgM-(Miltenyi, Bergisch Gladbach, Germany) or anti-IgG- (Promega, Madison, WI, USA) -HRP conjugated secondary antibodies diluted 1:4000 in Assay Diluent (BD Biosciences, San Diego, CA, USA). Afterwards, wells were washed five times with PBST, and 3,3′,5,5′-tetramethylbenzidine (TMB) substrate reagent was added to determine the antibody content by following the manufacturer's instructions. To measure the anti-αSyn specific antibodies in the serum samples, a similar protocol was followed but with some modifications. MaxiSorp (NUNC, Roskilde, Denmark) plates were coated with 1 µg of αSyn diluted in 100 µL of PBS in each well and incubated for 1 h at 37°C. After coating, washing, and blocking steps were performed as described above, and 1:40 diluted serum samples in Assay Diluent (BD Biosciences, San Diego, CA, USA) were added and incubated for 2 h at 37°C. Detection of IgM and IgG antibodies was performed as in the protocol described above. All samples were analyzed in duplicate.

To determine the IFN-γ and IL-10 levels, specific ELISA kits, namely Mouse IFN-gamma and Mouse IL-10 BD OptEIATM kits (BD Biosciences, San Diego, CA, USA), were used according to the manufacturer's instructions.

### Statistical analyses

Statistical analyses were performed by using the IBM SPSS Statistics 20 pack. For all parameters (T cell populations, antibody determinations, and cytokine measurements), the Kruskal–Wallis one-way analysis of variance was firstly performed to evaluate the existence of significant differences among the experimental groups. In order to determine the differences between groups and to obtain the *P* values, the non-parametric Mann–Whitney U test for two independent samples was performed. Each group consisted of 5–7 mice (*n* = 5–7). Statistically significant differences were those with *P* < 0.05.

## Results

### Preparation and characterization of a monomeric αSyn/Hsp70 complex

Based on our previous finding that recombinant human Hsp70 chaperone interacts with monomeric αSyn in vitro 40, we chose human Hsp70 to evaluate our immunization strategy with αSyn. In the preparation of the αSyn/Hsp70 complex to be used in biochemical studies as well as for immunization protocols, conditions favoring Hsp70/substrate-binding and therefore, the formation of the αSyn/Hsp70 complex, were chosen and applied for all sample preparations (i.e., αSyn/Hsp70 mixture, αSyn and Hsp70 proteins alone, and buffer/vehicle). For this, highly purified αSyn and Hsp70 in buffer (50 mM Tris pH 7.4, 150 mM KCl, 2 mM MgCl_2_) at a 1:1 molar ratio (or the corresponding protein amount for samples with αSyn or Hsp70 alone) were incubated at RT in the presence of 4 mM ATP for 2 h to induce the opening of the Hsp70 substrate binding pocket, and for a further 2 h with ADP added to a final concentration of 2.5 mM to favor the formation of the “high affinity” Hsp70/substrate complex 37.

**Figure 1:**
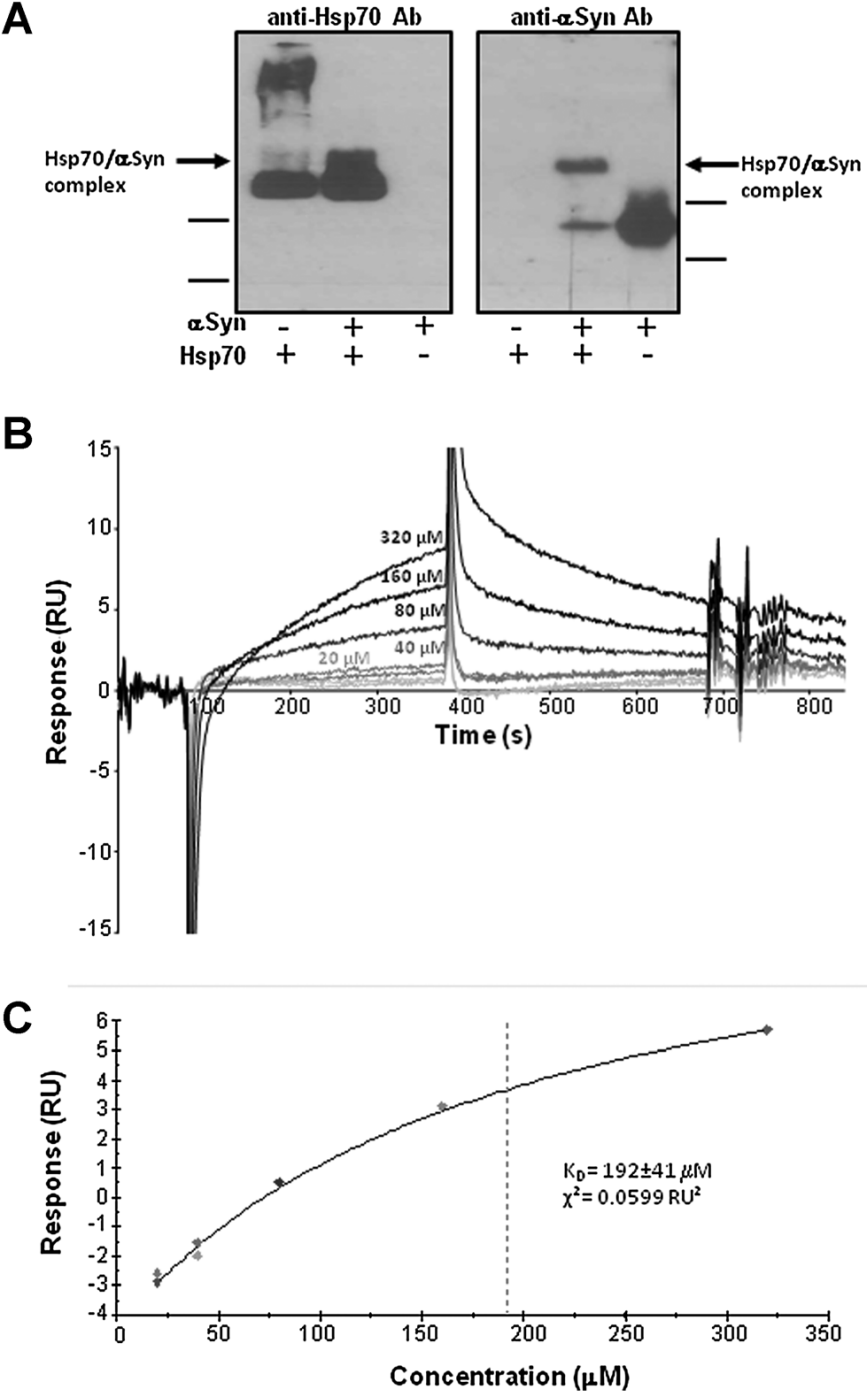
Characterization of the Hsp70-monomeric αSyn association and complex formation. Native PAGE and Western blot analysis of samples, by using anti-αSyn or anti-Hsp70 antibodies. In addition to the bands corresponding to Hsp70 and αSyn proteins, a highly overlapping, lower-mobility band can be seen in both Western blots, indicating the formation of an Hsp70/αSyn complex. Bovine serum albumin was used as a protein marker (indicated) (A). Binding of monomeric α-Syn to Hsp70 immobilized on a Biacore sensor chip. Sensorgrams illustrating dosage-dependent binding of α-Syn to 1173 RU of immobilized Hsp70 are represented (B). Steady-state concentration plot of α-Syn bound to Hsp70 5 seconds before the end of the analyte (α-syn) injection (C).

In order to evaluate the formation of an αSyn/Hsp70 complex, we subjected the incubated mixtures to native PAGE electrophoresis followed by Western blot with anti-Hsp70 and anti-αSyn specific antibodies (Fig. 1A). Indeed, we were able to detect a band shift in the lane corresponding to the αSyn/Hsp70 sample, as compared to the lanes loaded either with αSyn or with Hsp70 alone in both membranes labeled with either specific antibodies (Fig. 1A). This additional band, with apparently similar electrophoretic migration distance in both labeling assays, should correspond to a monomeric αSyn/Hsp70 complex.

To prove the interaction between monomeric αSyn and Hsp70 and further demonstrate the formation of an Hsp70/αSyn complex, we used surface plasmon resonance (Biacore, GE Healthcare). This allowed to monitor the interaction, in real time, between ATP-loaded Hsp70 and monomeric α-Syn through the covalent immobilization of the chaperone onto flow cell 2 (active cell) of the sensor chip and the injection of different α-Syn concentrations through both active and reference, flow cells. Signals from the active cell were corrected by subtracting signal from the reference one. Our data showed that the immobilized ligand (Hsp70) was able to specifically bind to the flowing analyte (monomeric αSyn) in an ATP-containing buffer (Fig. 1B). None of the sensorgrams could be fitted to a simple 1:1 Langmuir binding model in order to calculate kinetic parameters (*K*_a_ and *K*_d_), probably because the interaction between both molecules is more complex. Binding levels corresponding to a section of the curves 5 sec before the end of the injections were used to calculate the dissociation equilibrium constant (*K*_D_). The steady state binding levels against analyte concentration were plotted and fitted to a simple 1:1 fitting model available in the Biacore Evaluation Software. A good fit was obtained (*χ*^2^ value of 0.0599 RU^2^), with a calculated *K*_D_ value of 192 ± 41 µM (Fig. 1C), which should correspond to the (ATP)Hsp70/αSyn complex, that is in the “low-affinity” state 43. Therefore, it can be inferred that the *K*_D_ value of the (ADP)Hsp70/αSyn complex induced in our sample preparation for immunization, is lower than 190 µM.

### Restrained levels of anti-αSyn antibodies by αSyn/Hsp70-immunization

To determine the ability of Hsp70 to act as a paradigm “chaperone adjuvant” with αSyn, we designed and tested two immunization protocols in mouse with a combination of human αSyn and Hsp70 leading to the formation of a moderate-affinity complex. For this purpose, highly purified, endotoxin-free recombinant Hsp70 and αSyn proteins were used. Six to seven weeks old C57BL/6 male mice were injected subcutaneously at days 0 and 7, with either “low” (5 µg Hsp70 and/or 1.06 µg αSyn) or “high” (50 µg Hsp70 and/or 10.6 µg αSyn) protein doses, or buffer (vehicle), and maintaining a ca. 1:1 molar ratio for the mixture between αSyn and Hsp70. One week after the booster injection, mice were sacrificed and their spleen and whole blood were extracted for further analyses.

**Figure 2:**
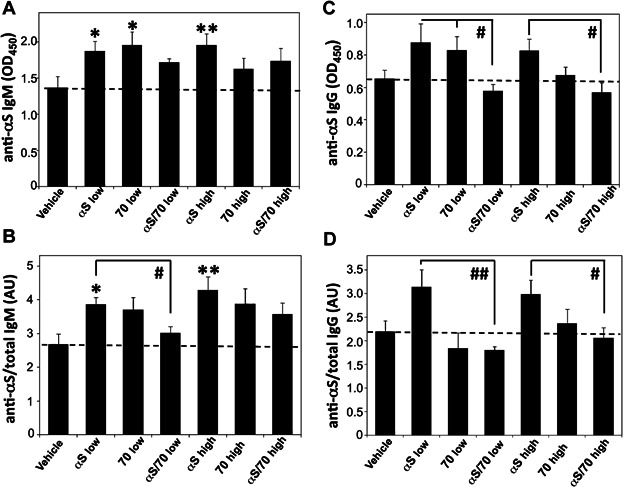
Humoral immune response characterization. Absolute anti-αSyn IgM (A) and relative anti-αSyn IgM (B) antibodies in serum from immunized mice. Absolute anti-αSyn IgG (C) and relative anti-αSyn IgG (D) antibodies in serum. The relative content of specific anti α-Syn IgM and IgG antibodies were calculated by dividing anti α-Syn IgM or IgG levels by the total corresponding antibody levels. AU: arbitrary units. Represented values are mean ± S.E.M. (*n* = 5–7). Asterisks correspond to statistically significant differences between one particular group and the “vehicle” group. Hash signs indicate statistically significant differences between different groups. *^/#^ *P* < 0.05, **^/##^ *P* < 0.01.

In order to evaluate the αSyn-specific humoral response resulting from the immunization protocol, we measured the levels of anti-αSyn as well as total IgM and IgG antibodies in serum (Fig. 2). For IgM, an increase in both the absolute anti-αSyn and relative anti-αSyn (calculated as anti-αSyn IgM/total IgM) antibodies was detected in the mouse groups immunized with both doses of αSyn (ca. 35–50%), and with “low” Hsp70 (ca. 40%, for absolute IgM levels), while this increase was virtually suppressed in the mouse group immunized with the αSyn/Hsp70 combination (Fig. 2A, B). A similar profile was observed for the measured IgG antibodies, with similar trends that did not reach statistical significance in absolute anti-αSyn and relative anti-αSyn (calculated as anti-αSyn IgG/total IgG) antibodies, for the “αSyn” experimental group (Fig. 2C, D). Also in this case, the anti-αSyn IgG antibody levels for both “αSyn/Hsp70” mouse groups were close to those in control mice, indicating a differential “restrain” effect arising from immunization with the αSyn/Hsp70 combination (Fig. 2B, D).

### The regulatory (Treg) and effector (Teff) T cell contents are affected by immunization

Because of the pivotal participation of CD4^+^ T cells in the development of synucleinopathies and other misfolding neurodegenerative diseases associated to chronic inflammation, we firstly compared the impact of immunizing mice with vehicle, αSyn, Hsp70, or the αSyn/Hsp70 combination, on the CD4^+^ cell populations within total splenocytes (Supporting Information Fig. S1). Splenocytes were isolated from immunized and sacrificed mice. The content of CD4^+^ cells in total splenocytes as analyzed by flow cytometry showed no significant differences between mouse groups (data not shown).

**Figure 3:**
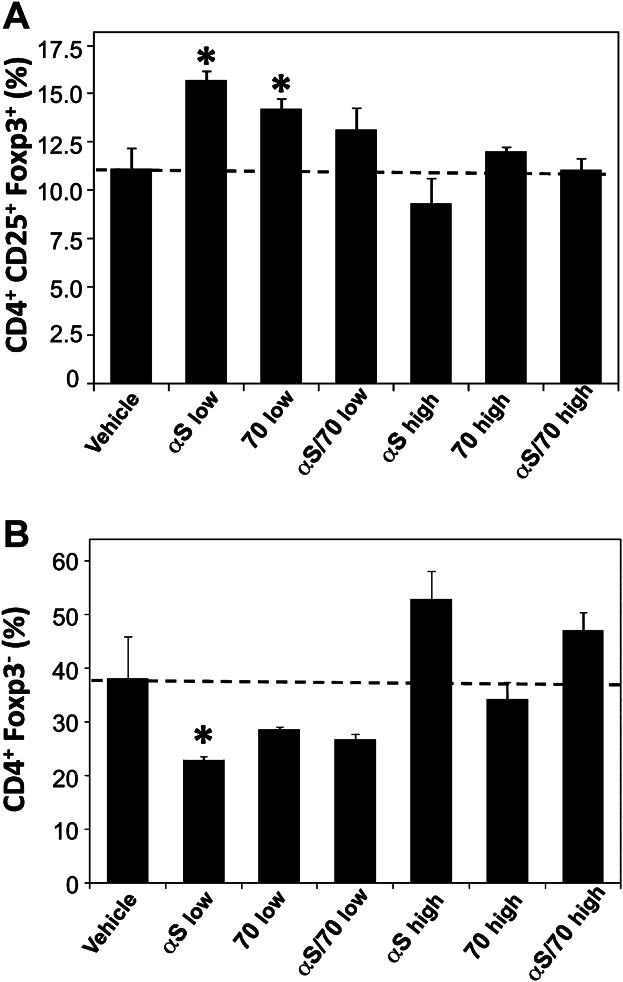
Treg and Teff cell populations determination. Percentage of Treg lymphocytes calculated as the percentage of cells positive for CD4, CD25, and Foxp3 staining among the CD4^+^ lymphocyte population (CD4^+^CD25^+^Foxp3^+^) (A). Percentage of Teff lymphocytes calculated as the percentage of cells with positive staining for CD4 and negative for Foxp3 among the CD4^+^ lymphocyte population (CD4^+^Foxp3^−^) (B). Represented values are mean ± S.E.M. (*n* =5–7). Asterisks correspond to statistically significant differences between one particular group and the “vehicle” group. * *P* < 0.05, ** *P* < 0.01.

Next, in order to compare the regulatory T (Treg) cell population contents between the different mouse groups, we quantitated the CD4^+^CD25^+^Foxp3^+^-labeled cells in the isolated splenocytes (Fig. 3A and Supporting Information Fig. S1). Our results showed that at “low” concentrations of immunogen, both the groups immunized with αSyn and, to a lower degree, Hsp70, produced a significant increase in the Treg percentage (15.7 ± 0.5% and 14.3 ± 0.5%, respectively), as compared to the mouse group immunized with vehicle (control) (11.1 ± 1.0%). However, this effect was not observed using the αSyn/Hsp70 mixture (13.1 ± 1.2%), further indicating a differential effect of this combination (Fig. 3A). The mice immunized with high protein doses, on the other hand, did not show any differences in the percentages of Treg cells compared to the control mice.

Finally, in order to compare the content of the non-regulatory/effector T (Teff) cell population in the isolated splenocytes from the different mouse groups, we assayed the percentage of Foxp3^−^ (Fig. 3B and Supporting Information Fig. S1). In this case, a significant reduction was measured in the CD4^+^Foxp3^−^ cell population content for the mouse group immunized with a “low” dose of αSyn (22.8 ± 0.7%) relative to the group injected with “vehicle” (38.1 ± 7.8%) (Fig. 3B).

### Differential immunoreactivity of αSyn- versus LPS-pulsed splenocytes from immunized mice

**Figure 4:**
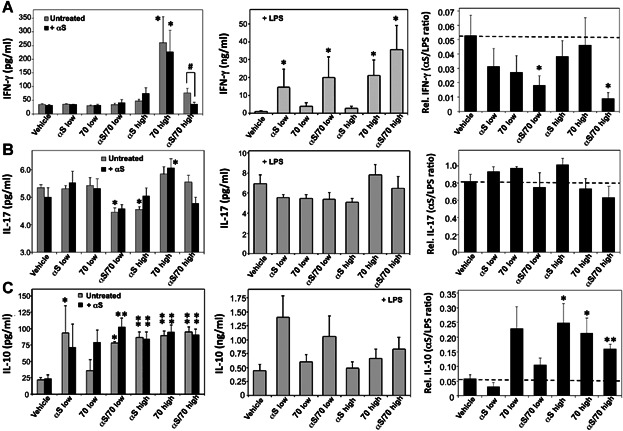
Cytokine release profile of splenocytes isolated from immunized mice. IFN-γ (A), IL-17 (B), and IL-10 (C) levels were measured in supernatants from cultured splenocytes isolated from immunized mice 24 h after treatment with αSyn (20 µg/mL) or medium alone (left column), or with LPS (0.5 µg/mL) (center column). All cytokines were measured by ELISA. Pairwise αSyn/LPS ratios (right column) were calculated by dividing the cytokine levels elicited by αSyn (pg/mL) by the corresponding cytokine response to LPS (pg/mL) for each mouse. The detection limit for the IL-10/IFN-γ and IL-17 assay kits were <30, and 4 pg/mL, respectively. Represented values are mean ± S.E.M. (*n* = 5–7). Asterisks correspond to statistically significant differences between one particular group and the “vehicle” group. Hash signs indicate statistically significant differences between different groups. *^/#^ *P* < 0.05, **^/##^ *P* < 0.01.

Next, as a way to monitor possible αSyn-specific, hyper-/hypo-immune responses, we measured cytokine release profiles of cultured splenocytes isolated from immunized mice after sacrifice. To this end the cultured cells from the different experimental groups were pulsed either with 20 µg/mL αSyn (to model the antigen specific immunoreactivity in vitro) or 1 µg/mL LPS (to model non-specific/polyclonal immunoreactivity in vitro) or, alternatively, medium alone to determine the basal secretion levels, which corresponds to a non-stimulated control (Fig. 4). In particular, IFN-γ and IL-17 as well as IL-10, were measured in the supernatant collected after 24 h of incubation. Our results show that splenocytes from mice immunized with a “high” dose of Hsp70 produced higher basal levels of IFN-γ (260 ± 97 pg/mL), as compared to splenocytes from mice immunized with vehicle (Fig. 4A, left panel). On the other hand, stimulation of cultured splenocytes with LPS induced higher IFN-γ secreted levels in cells from mice immunized with “low αSyn” (15 ± 10 ng/mL) and the “low αSyn/Hsp70” complex (20 ± 12 ng/mL), as well as with “high Hsp70” (21 ± 9 ng/mL), as compared to the group immunized with vehicle (Fig. 4A, central panel). However, the largest induction of IFN-γ was observed for cells isolated from mice immunized with the “high αSyn/Hsp70” combination (36 ± 14 ng/mL) (Fig. 4A, central panel). Notably, this higher general reactivity in the case of the high dose of “αSyn/Hsp70” did not correlate with that elicited by αSyn pulsing, as lower than basal levels of IFN-γ were measured as a result of αSyn-pulsing of splenocytes for this group (Fig. 4A, left panel). Interestingly, lower values of secreted IL-17 levels for the “low αSyn/Hsp70” (4.5 ± 0.17 pg/mL) and “high αSyn” (4.6 ± 0.11 pg/mL) groups were measured as compared to the group immunized with vehicle (5.4 ± 0.11 pg/mL), while an increase for the “high Hsp70” group (6.1 ± 0.34 pg/mL) relative to the control (5.0 ± 0.35 pg/mL was detected upon αSyn-pulsing of splenocytes (Fig. 4B, left panel). On the other hand, no significant differences in secreted IL-17 levels as a result of LPS-stimulation of splenocytes were detected (Fig. 4B, central, panel).

Regarding the immunomodulatory-linked cytokine IL-10, splenocytes from all groups, except for the “low Hsp70” group, showed higher basal levels of IL-10 as compared to splenocytes from mice immunized with vehicle (three- to fivefold higher) (Fig. 4C, left panel). Pulsing splenocytes with αSyn did not significantly alter this profile, except for the “low Hsp70” experimental group. Stimulation with LPS produced more variability in IL-10 levels between the various groups, although none of them reached statistical significance as compared to the “vehicle” group (Fig. 4C, central panel).

In order to normalize the αSyn-specific response to the general splenocyte reactivity, we also analyzed the αSyn/LPS ratio for the three cyokines measured (Fig. 4A, right panel). A clear downregulation in the relative IFN-γ secretion levels upon challenge with αSyn could be observed for mice immunized with the αSyn/Hsp70 combination at both doses (three- to fivefold lower) and essentially unaltered relative IL-17 levels (Fig. 4A, B, right panel). Conversely, significant increases in the relative IL-10 levels upon αSyn challenge were seen for the “high αSyn” and “high Hsp70” experimental groups (fivefold higher), in addition to “high αSyn/Hsp70” combination (threefold higher) (Fig. 4C, right panel). Taken together, our results suggest that highly reactive splenocytes that are hypo-responsive toward αSyn and which display high IL-10 basal secreting levels, have been uniquely generated by immunization of mice with the αSyn/Hsp70 combination.

### IFNγ and IL-10 cytokine levels in serum indicate a shift toward an immunomodulatory response in αSyn/Hsp70-immunized mice

**Figure 5:**
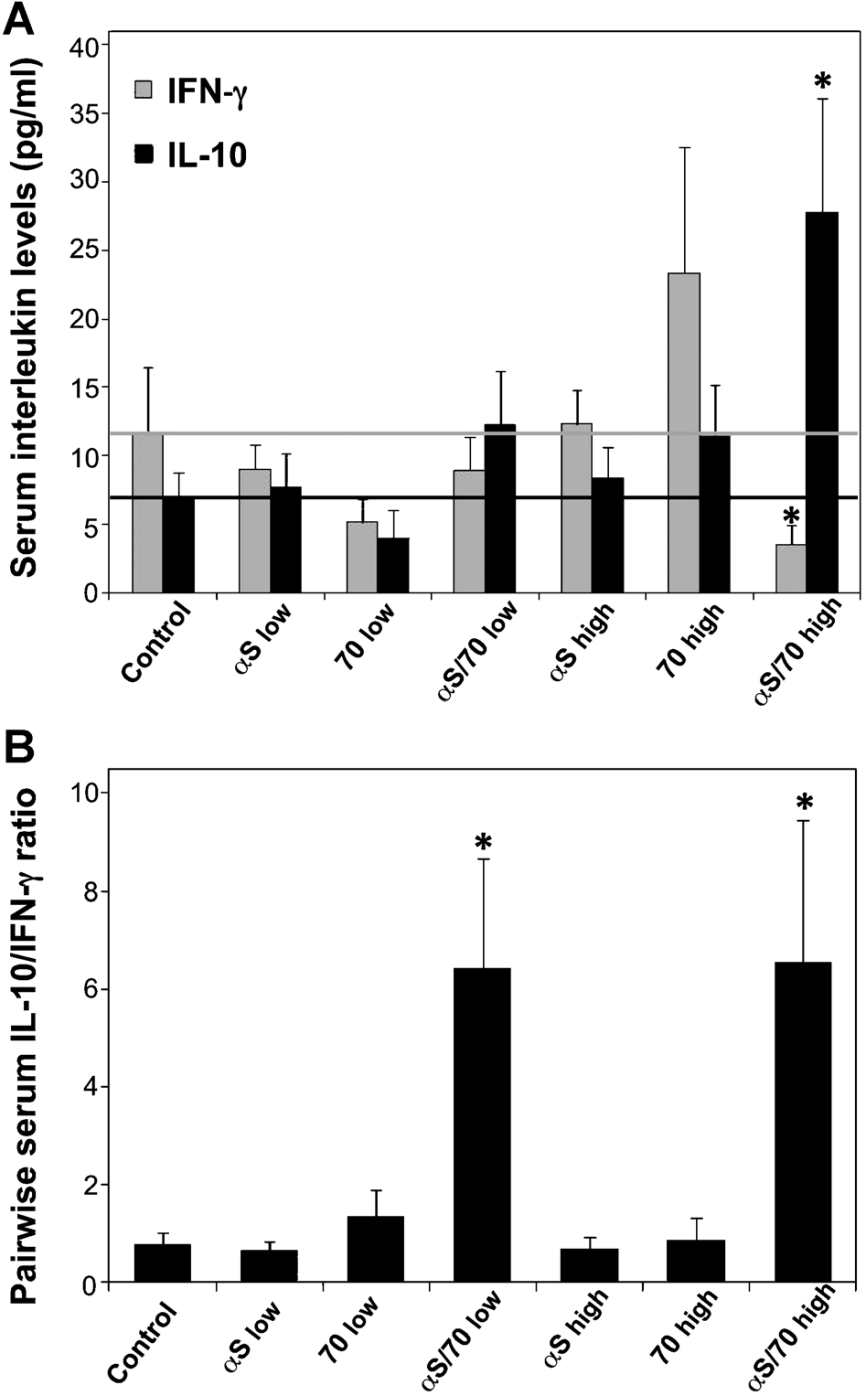
IFN-γ and IL-10 serum levels in immunized mice. IFN-γ (gray) and IL-10 (black) levels were measured in mouse sera one week after the end of the immunization protocol, by ELISA (A). Pairwise IL-10/IFN-γ ratios were calculated by dividing the measured IL-10 cytokine level by the measured IFN-γ cytokine level for each mouse (B). The detection limit for the IL-10 and FN-γ assay kits were <30 pg/mL, Values are mean ± S.E.M. (*n* = 5–7). Asterisks correspond to statistically significant differences between one particular group and the “vehicle” group. * *P* < 0.05, ** *P* < 0.01.

To evaluate the biological relevance in vivo of immunization with the αSyn/Hsp70 complex, we assayed the levels of IFN-γ and IL-10 in serum obtained from immunized mice, one week after the booster (Fig. 5). While virtually all mouse groups showed no significant alterations in serum IFN-γ levels as compared to the vehicle group (12 ± 5 pg/mL), the only significant change in this cytokine levels corresponded to a threefold reduction for the “high αSyn/Hsp70” combination (3.6 ± 1.4 pg/mL) (Fig. 5A). Furthermore, the only significant change in serum IL-10 levels observed corresponded to a fourfold increase for the mouse group immunized with “high αSyn/Hsp70” (28 ± 8 pg/mL) as compared to mice immunized with “vehicle” (7.0 ± 1.8 pg/mL) (Fig. 5A). Finally, we analyzed the pairwise serum IL-10/IFN-γ ratio (i.e., the IL-10/IFN-γ measured levels for each mouse) and compared the different experimental groups. Remarkably, while the IL-10/IFN-γ ratio for the “αSyn” and “Hsp70” groups remains close to that observed for the “vehicle” group, immunization of mice with the “αSyn/Hsp70” combination at both doses produces a ca. eightfold increase in this ratio (Fig. 5B). This unique effect of the “αSyn/Hsp70” combination is evidenced by the shift in the serum IL-10/IFN-γ relative levels, from a pro-inflammatory, toward an immunomodulatory, profile (Fig. 5B).

## Discussion

Over the last few years, immunotherapy has become an expanding subject of study as a novel approach for the treatment of neurodegenerative amyloid or ‘misfolding’ diseases, which remain essentially incurable. Accumulated evidence highlights the central role in neurodegenerative disorders of an uncontrolled cellular-mediated response that could promote microglial activation and neuroinflammation, and ultimately lead to neurodegeneration 44,45. In the present work we have combined the aggregating, IDP protein αSyn and the full-length and functional Hsp70 chaperone to promote the formation of an αSyn/Hsp70 complex in vitro, and used it to vaccinate naive mice in the absence of added adjuvants, to evaluate the resulting immune response.

Our findings reveal that, while increased levels of anti-αSyn IgM and IgG antibodies were produced by immunization with αSyn and Hsp70 proteins alone, no such effect was observed for the group vaccinated with the αSyn/Hsp70 combination, as compared to the control group. This unique effect of αSyn/Hsp70 immunization to produce a restrained anti-αSyn Ab response might be beneficial in the context of PD as it has been suggested that anti-αSyn antibodies are involved in the pathogenesis of the inherited form of the disease 46–48. Moreover, this feature could be especially positive in the case of well-established autoimmune-related neurodegenerative diseases such as Multiple Sclerosis (MS) or Acute Motor Axonal Neuropathy, where elevated levels of specific IgM and IgG antibodies directed against certain self antigens have been shown to contribute to disease onset and progression 49–53.

A similar trend was seen for the Treg and Teff cell populations, in which the increments and reductions, respectively, elicited by immunization with αSyn and Hsp70 alone were restrained as a result of vaccination with the αSyn/Hsp70 complex, again indicating a differential effect of the combined proteins and the formation of an immunofunctional complex. This “buffering” of the regulatory and non-regulatory/effector T cell content toward basal levels as a result of immunization with the αSyn/Hsp70 complex – as opposed to immunization with both proteins separately – could be potentially beneficial as it indicates a capacity of the αSyn/Hsp70 immunization to restore or maintain the Treg/Teff equilibrium in a disease, or pre-onset, scenario.

Our results with cultured splenocytes from immunized mice and subsequent challenge with αSyn or LPS, show clearly that the αSyn-specific response is suppressed in the case of vaccination with the αSyn/Hsp70 complex when compared to vehicle-injected mice, as indicated by lower αSyn-specific IFN-γ and IL-17 relative secretion levels. Even though other cells types present within splenocytes could also respond to LPS stimulation, in addition to T cells – such as B cells or dendritic cells – this result may reflect a lower Th1/(Th17) response (a T cell-mediated response involving the liberation of IFN-γ and IL-17) toward αSyn as a result of immunization with the αSyn/Hsp70 complex. In addition, we found that this change is accompanied by higher basal levels of secreted IL-10 that are not significantly altered when challenged with αSyn.

Finally, we found that immunization with the αSyn/Hsp70 complex uniquely produced changes in the cytokine levels in serum, consisting of a threefold reduction of IFN-γ and a fourfold increase of IL-10 absolute levels, as well as a sixfold increase in the pairwise IL-10/IFN-γ ratio, as compared to vehicle-injected mice. These results clearly demonstrate that the αSyn/Hsp70 combination produces a shift in the peripheral immunity toward a modulatory – and presumably protective – phenotype. This effect could potentially be highly beneficial for treatment against misfolding diseases and other neurodegenerative disorders, as the Th1/Th17 response has been linked to microglia polarization and/or maintenance toward an M1 (classical) pro-inflammatory phenotype 54,55, which in turn has been shown to enhance neurodegeneration in PD 17,56,57.

Overall, our results show that human Hsp70 chaperone acts as a cell immunity adjuvant in combination with full-length αSyn, producing a shift in the immune response characterized by a restrained αSyn-specific humoral immunity coupled to a modulatory/protective phenotype, in immunized naive mice (Scheme 6). This shift toward an immunomodulatory phenotype cannot, in principle, be attributed to an increase in the Treg cell content, as similar levels of Treg cells from spleen were measured in the αSyn/Hsp70-immunized mice as compared to the control group. Therefore, either a different quality of Treg cells, or the engagement of other cell types such as NKT cells, B lymphocytes or even dendritic cells, could potentially be involved in this particular phenotype.

**scheme:**
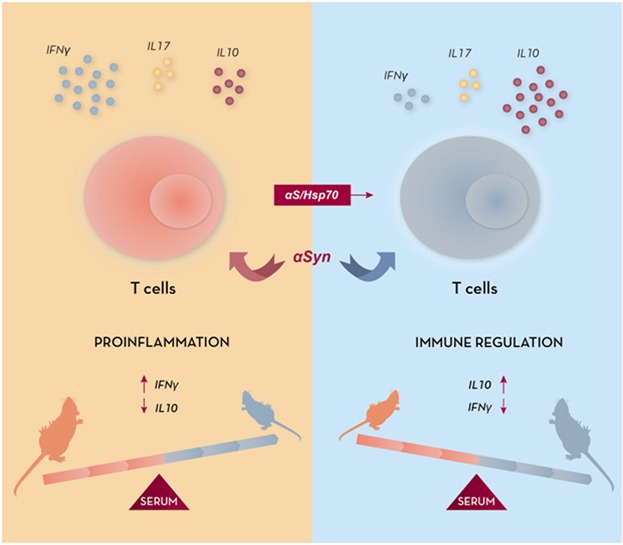
Immunization with α-Syn/Hsp70 produces a shift from a proinflammatory profile toward an immunomodulatory profile. Immunization of mice with the αSyn/Hsp70 complex in the absence of added adjuvant generates a unique response consisting of highly reactive splenocytes toward a non-specific/polyclonal insult which display higher IL-10 basal secreting levels, and which are hypo-responsive toward αSyn. In vivo, immunization with αSyn/Hsp70 produces a restrained anti-αSyn Ab humoral response, as compared to immunization with αSyn alone. This effect is accompanied by unique changes in IL-10 and IFN-g cytokines serum levels, consisting of higher immunomodulatory IL-10 and lower Th1-linked IFN-γ, and producing clear-cut shifts in the serum IL-10/IFN-γ relative levels. Overall, immunization with the “αSyn/Hsp70” combination generates a shift in cellular immunity from a proinflammatory profile, toward an immunomodulatory phenotype.

With the aim of minimizing the potential adverse effects of immunization, such as exacerbated inflammation or autoimmunity, two αSyn-based vaccination approaches designed to bypass the antigen-specific cellular immunity completely, while promoting a strong antigen-specific humoral response, have been recently reported 23,24. In this work we describe an alternative strategy that is based on the use of a combination of Hsp70 and αSyn to produce a restrained anti-αSyn humoral response coupled to an immunomodulatory phenotype. We propose that such a combined response might simultaneously tackle two aspects of PD pathobiology at the onset and progression stages: firstly, an immunoregulatory phenotype in the periphery is expected to exert a neuroprotective effect by communicating and interacting with chronically activated microglia and other immunocompetent cells in the CNS, and therefore counteracting neuroinflammation. Secondly, it might prevent the development of potentially detrimental polyclonal antibody responses against αSyn in an αSyn overexpression or aggregation scenario, as opposed to a specific antibody response directed toward toxic oligomeric species or epitopes, of αSyn.

Furthermore, we suggest that the exploitation of the differential immune response elicited by specific combinations of aggregating proteins with immunoactive and functional chaperones, or by certain HSP/aggregating protein complexes, could be an effective means of suppression of the uncontrolled pro-inflammatory environment associated to neurodegeneration in amyloid and other misfolding disorders.

## Supporting Information

**Figure S1.** Representative flow cytometry plots for Treg and Teff cell populations.
