# Supplementary figures and images for: Chaperoned amyloid proteins for immune manipulation: α-Synuclein/Hsp70 shifts immunity toward a modulatory phenotype
Source: Immun Inflamm Dis. 2014 Dec 5;2(4):226–38. doi: 10.1002/iid3.39 (PMC4386917; doi:10.1002/iid3.39)

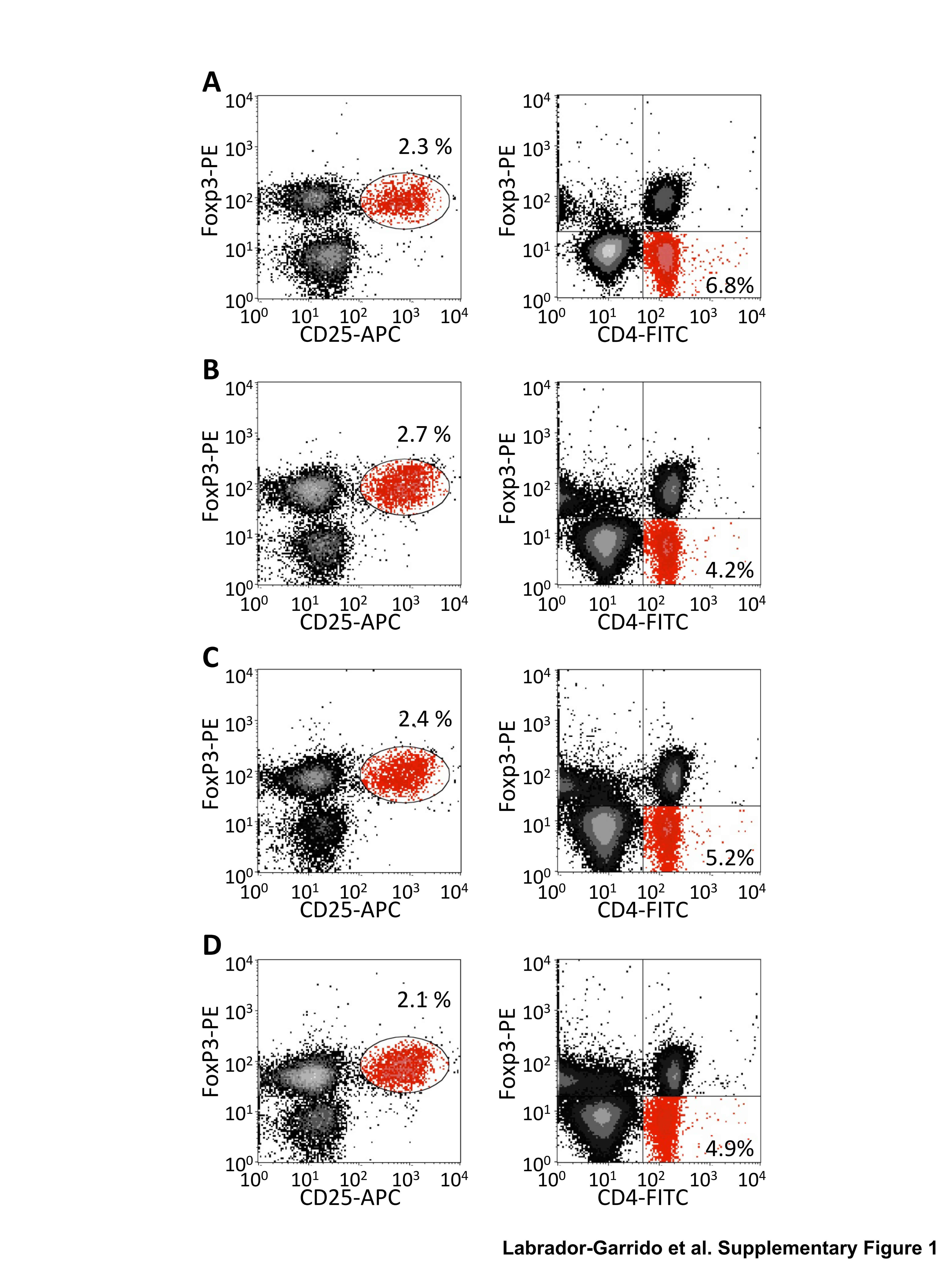

Supplement: Supplementary file 1 [file iid30002-0226-sd1.jpg]
